# 血红蛋白M病Iwate型合并弥漫大B细胞淋巴瘤1例

**DOI:** 10.3760/cma.j.issn.0253-2727.2023.07.018

**Published:** 2023-07

**Authors:** 善炜 叶, 克峰 沈, 敏 肖, 沛凌 张, 世媛 张, 婷 邓, 亮 黄, 晓曦 周

**Affiliations:** 1 华中科技大学同济医学院附属同济医院，武汉 430030 Tongji Hospital, Tongji Medical College, Huazhong University of Science & Technology, Wuhan 430030, China; 2 重庆市第五人民医院，重庆 400062 Department of Hematology, The Fifth People's Hospital of Chongqing, Chongqing 400062, China

患者，女，福建福州人，自幼面部及四肢皮肤呈暗灰色，口唇、舌及甲床明显紫绀，但发育未见明显异常，无缺氧表现，剧烈运动和体力劳动不受限。其父亲、弟弟和女儿均有类似表现。患者自述分别于1995年行剖宫产术，1997年行胆囊切除术，2020年行阑尾切除术，过程顺利，未输血及未表现出缺氧或无法耐受麻醉等情况。

至患者56岁时，因无明显诱因腹痛，伴低热、盗汗就诊，经B超引导下腹腔淋巴结穿刺活检，诊断为弥漫大B细胞淋巴瘤（非生发中心型、Ⅲ期B组、TP53突变）。经R-CHOP（利妥昔单抗、环磷酰胺、多柔比星、长春新碱和泼尼松）方案化疗4个周期，疗效评估为部分缓解（PR）；后给予R-DA-EPOCH（利妥昔单抗+剂量调整的依托泊苷+泼尼松+长春新碱+环磷酰胺+表柔比星）方案化疗3个周期，同时口服泽布替尼，达到完全缓解；再行1个疗程利妥昔单抗治疗，后定期门诊随访。7个月后发现疾病进展，外院予Gemox（吉西他滨+奥沙利铂）联合西达苯胺及沙利度胺方案化疗2个周期；奥妥珠单抗+异环磷酰胺+依托泊苷+塞利尼索方案化疗1个周期；奥妥珠单抗+塞利尼索方案化疗1个周期。疗效不佳，来我院进一步诊疗。

该患者为多线治疗失败的弥漫大B细胞淋巴瘤患者，淋巴瘤细胞表达CD19^−^CD22部分^+^、CD79b^+^，选用靶向CD79b的泊洛妥珠单抗+DHAP（地塞米松+顺铂+阿糖胞苷）进行挽救化疗，并动员造血干细胞，为自体造血干细胞移植做准备。

入院查体：神志清楚，发育正常。面部及四肢皮肤呈暗灰色，口唇、舌及甲床明显紫绀，无杵状指。心肺听诊正常，肝脾未触及肿块，无肾区叩击痛。测指脉氧，血氧饱和度仅76％，结合先天口唇指甲发绀问题，进一步行动脉血气分析：酸碱度7.419，二氧化碳分压35.6 mmHg，氧分压150 mmHg，实际碳酸氢根22.6 mmol/L，标准碳酸氢根23.2 mmol/L，全血剩余碱−1.1 mmol/L，细胞外液剩余碱−1.3 mmol/L，氧饱和度75.1％；全外显子测序：HBA2:c.262>T（p.H87Y）胚系杂合突变，即Hb M-Iwate突变体。同时检测其女儿全外显子测序，也发现同样突变。

患者入院HGB 109 g/L，美国东部肿瘤协作组体能状况评分（ECOG评分）1分。经化疗后出现骨髓抑制，HGB最低76 g/L。自HGB低于80 g/L开始，患者体能状况明显下降，ECOG 4分，全天卧床，无法下床，伴胸闷，吸氧（2～6 L/min）缓解不明显，头晕头痛，食欲明显减退，依赖静脉营养。予以输血支持后症状可得到明显改善。

讨论：本病例为中年患者，化疗后骨髓抑制期，从轻度贫血进展为中度贫血时即表现出异常严重的贫血症状，包括体能状态下降（ECOG评分4分），胸闷、头晕头痛、食欲下降等。这些严重症状，在非急性失血或溶血的中年中度贫血患者中不常见。尽管Hb M-Iwate突变形成的异常亚基使氧亲和力降低，但该突变多为杂合突变，因此异常亚基含量一般较低，故患者一般生理状态下日常生活和体力劳动均不受影响且血红蛋白和红细胞都在正常参考值范围内。本组患者因接受挽救性化疗导致骨髓抑制，在红细胞生成减少并导致低血红蛋白的情况下，还存在由于Hb M-Iwate突变形成的异常血红蛋白，导致携氧效率进一步下降，使患者出现与血红蛋白值及下降速率不相符的严重贫血症状。该患者血气分析显示氧饱和度低，可能是由于血红蛋白数量减少（贫血）及质量异常（Hb M-Iwate突变导致血红蛋白低氧亲和力），使得氧合血红蛋白形成减少，大量氧气以物理形式溶解在血浆中，因此氧分压增高。综上所述，在合并血红蛋白M病Iwate型的肿瘤患者化疗的过程中，特别是进行易出现骨髓抑制的化疗时，需要密切关注患者贫血症状，采用更为积极的输血策略，更多地依据临床症状而不是血红蛋白数值来决定输血指征。

